# Identification and Elucidation of the Protective isomiRs in Lung Cancer Patient Prognosis

**DOI:** 10.3389/fgene.2021.702695

**Published:** 2021-09-13

**Authors:** Fu-Mei Hsieh, Su-Ting Lai, Ming-Fong Wu, Chen-Ching Lin

**Affiliations:** Institute of Biomedical Informatics, National Yang Ming Chiao Tung University, Taipei, Taiwan

**Keywords:** miRNA, isomiR, lung adenocarcinoma, survival analysis, miR-181a-3p, miR-21-5p

## Abstract

MicroRNAs (miRNAs) are approximately 20–22 nucleotides in length, which are well known to participate in the post-transcriptional modification. The mature miRNAs were observed to be varied on 5′ or 3′ that raise another term—the isoforms of mature miRNAs (isomiRs), which have been proven not the artifacts and discussed widely recently. In our research, we focused on studying the 5′ isomiRs in lung adenocarcinoma (LUAD) in The Cancer Genome Atlas (TCGA). We characterized 75 isomiRs significantly associated with better prognosis and 43 isomiRs with poor prognosis. The 75 protective isomiRs can successfully distinguish tumors from normal samples and are expressed differently between patients of early and late stages. We also found that most of the protective isomiRs tend to be with downstream shift and upregulated compared with those with upstream shift, implying that a possible selection occurs during cancer development. Among these protective isomiRs, we observed a highly positive and significant correlation, as well as in harmful isomiRs, suggesting cooperation within the group. However, between protective and harmful, there is no such a concordance but conversely more negative correlation, suggesting the possible antagonistic effect between protective and harmful isomiRs. We also identified that two isomiRs miR-181a-3p|-3 and miR-181a-3p|2, respectively, belong to the harmful and protective groups, suggesting a bidirectional regulation of their originated archetype—miR-181a-3p. Additionally, we found that the protective isomiRs of miR-21-5p, which is an oncomiR, may be evolved as the tumor suppressors through producing isomiRs to hinder metastasis. In summary, these results displayed the characteristics of the protective isomiRs and their potential for developing the treatment of lung cancer.

## Introduction

MicroRNAs (miRNAs) are a class of small non-coding RNAs (ncRNA) of approximately 22 nts in length encoded in the genome of plants and animals, which have been found to regulate gene expression in the post-transcriptional level (Lee et al., 1993; Reinhart et al., 2000). MiRNAs were found to bind on the 3′ untranslated region (UTR) of target mRNA, which was called miRNA response element (MRE), and usually repress the target mRNA expression (Lai, 2002). Typically, miRNA is first transcribed to primary miRNA (pri-miRNA) by RNA polymerase II and folded to a hairpin structure. Then, the Drosha-DGCR8 complex binds on the pri-miRNA to prune the stem and produce miRNA precursor (pre-miRNA), which is approximately 60 nts. The pre-miRNAs are further exported into the cytoplasm. In the cytoplasm, the hairpin loop of pre-miRNA is trimmed by Dicer to generate a paired miRNA duplex, each approximately 22 nts. Either strand of the paired miRNA duplex can further bind to the Argonaute protein (AGO) (Olina et al., 2018), form a silencing complex (RISC), and then regulate, usually repress, the target gene expression (Bartel, 2018).

“Lung cancer has been noticed as one of the leading causes of death in cancers in the United States and around the world” (Dela Cruz et al., 2011). Besides, lung cancer has a poor prognosis and thus is worth noticing its progression in treatment, biomarkers, etc. The most common type is non-small cell lung cancer (NSCLC), which takes 85% of lung cancer. NSCLC can be further classified into three subtypes: adenocarcinoma, squamous cell carcinoma, and large cell carcinoma. In this study, we mainly focused on lung adenocarcinoma (LUAD), which comprises 40% of all lung cancer (Zappa and Mousa, 2016). MiRNAs are involved in many mechanisms in cells, such as immune responses (Sun et al., 2013; Xu et al., 2014) and tumor progression. They can be tumor suppressors and oncogenes (Esquela-Kerscher and Slack, 2006). More importantly, because of their ubiquitous characteristic, miRNAs are widely detected and found to contribute to cancer treatment (Berindan-Neagoe et al., 2014; Shah et al., 2016). Recent studies have provided insight into the role of miRNAs in tumorigenesis and predicted miRNAs as biomarkers in lung cancer (Azizi et al., 2021; Zhong et al., 2021). Additionally, the functions of miRNA in lung cancer have been widely reported (Iqbal et al., 2019; Wu et al., 2019).

With the development of sequencing technology, the mature miRNAs were observed to be varied on 5′ or 3′ that raise the isoforms of mature miRNAs (isomiRs) (Morin et al., 2008; Guo et al., 2016). Notably, these isomiRs were indicated to be mainly generated from imprecise cleavage or shifting by Drosha and Dicer rather than the degradation during sample preparation for sequencing (Lee et al., 2010; Neilsen et al., 2012). Additionally, some isomiRs can be generated by non-template nucleotide addition, such as adenylation at the 3′-end (Lu et al., 2009). Accordingly, the identification of isomiRs extends the functional significance and variance of miRNA regulation (Mercey et al., 2017). IsomiRs can generally be categorized into three types: 3′ isomiR, 5′ isomiR, and polymorphic isomiR. MiRNA can interact with its mRNA target by Watson–Crick pairing typically through the strong connection of “seed region,” which is located on 2-7mer (or 8mer) of 5′ miRNA and guided mRNA targeting (Bartel, 2009; Tan et al., 2014). Therefore, the generation of 5′ isomiRs could alternate the seed region and affect the downstream regulation and functional roles of miRNAs. Recently, the isomiRs have been discovered to show different expression levels among disease subtypes, gender differences (Loher et al., 2014; Guo et al., 2016). The existence of isomiRs may also help to distinguish the cancer type (Telonis et al., 2017). Also, isomiRs may play the dominant miRNA in certain tissues or even become the canonical miRNAs through evolution (Tan et al., 2014). The miRNA regulation relies on miRNA–mRNA complementary binding. The presence or even domination of isomiR indicates the concern of nucleotides alternation in miRNA regulation, therefore elevating the urgency of clarifying the variance of miRNA regulation derived from isomiRs.

## Materials and Methods

### Collection and Preprocessing of miRNA and mRNA Expression Profiles

The miRNA expression profiles ‘‘isoforms.quantification.txt’’ were obtained from The Cancer Genome Atlas (TCGA)-GDC portal^1^, which recorded the coordinate information and expression level miRNAs and their isomiRs. We initially downloaded 567 LUAD samples. For data cleaning, 87 were removed as they contain “annotation.txt,” which may be problematic. Also, three duplicated files from the same participant (TCGA-44-6775) and two “Recurrent Solid Tumor” files were skipped. Finally, 475 LUAD miRNA expression files, which contain 442 primary solid tumors and 33 solid tissue normal, were kept. Meanwhile, the RNA-seq data “htseq.counts” were also downloaded. After data cleaning as the same criteria with miRNA expression profiles, 480 RNA-Seq samples (46 normal, 434 tumor) remained. Finally, we kept a total of 444 RNA-Seq samples (14 normal, 430 tumor) that match the isomiR expression profiles for the analysis of miRNA-regulated target genes.

### The Nomenclature of isomiRs

**FIGURE 1:**
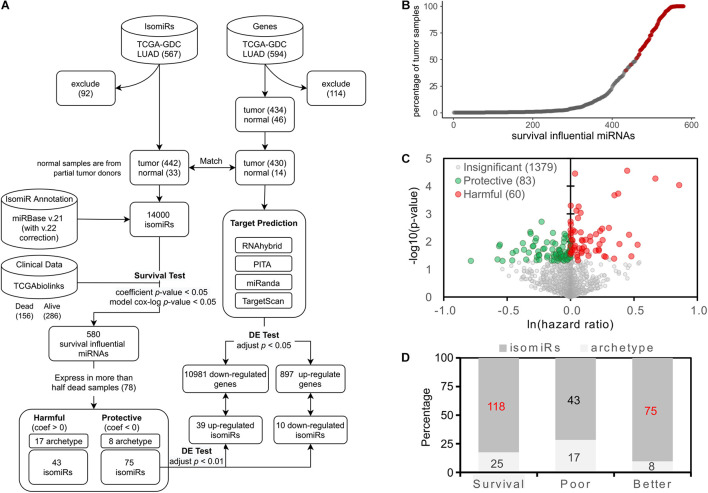
Identification of the survival influential miRNAs. **(A)** The overall workflow of identifying the survival influential miRNAs. **(B)** Percentage of samples in which the survival influential miRNAs are expressed. The *x*-axis lists the miRNAs, which are significantly associated with patients’ prognoses, ordered by the number of samples in which they are expressed. The red dots are those survival influential miRNAs expressed in at least 50% of dead samples. **(C)** Volcano plot for survival influential miRNAs. The *x*-axis is the natural log of the hazard ratio of miRNAs; the *y*-axis shows the corresponding significance. The miRNAs with significant influence on patient prognosis are colored by green (protective) and red (harmful). **(D)** The prevalence of survival influential isomiRs. The survival category indicates all the survival influential miRNAs, i.e., contains poor and better prognoses. Numbers labeled red indicate a significantly overrepresented proportion of isomiRs.

In our research, the isomiRs are defined by the relative 5′ start site of archetype miRNA. Since the miRNA annotation of TCGA data was according to the miRBase V21, and the archetype miRNA coordinate “hsa.gff3” was initially downloaded from miRBase (V21 from http://www.mirbase.org/) (Kozomara and Griffiths-Jones, 2014; Kozomara et al., 2019). The nomenclature of isomiRs was defined by subtracting the 5′ start site coordinates of the archetype miRNA, which are recorded in “hsa.gff3,” and each corresponding isomiR, which is recorded in “isoforms.quantification.txt,” followed by previous studies (Telonis et al., 2015, 2017). For example, “MIMAT0000062|-2” and “MIMAT0000062|4” are isomiRs of the archetype “MIMAT0000062” with respectively two upstream and four downstream nucleotides shifting of the archetype. Additionally, we denoted the archetypes as the miRNA with zero shift; for example, the archetype of MIMAT0000062 is named as MIMAT0000062|0. Positive or negative strands and location on chromosomes were all considered in different subtraction cases. Herein, we use miRNAs to represent the set of archetype miRNA and isomiRs. Furthermore, the miRBase has been updated to V22 (Kozomara et al., 2019); we then updated the annotation of miRNAs and isomiRs in TCGA dataset for the analysis in this study. The overall workflow of this study is depicted in Figure 1A.

### Identification of Survival Influential and Differentially Expressed isomiRs

In this study, we applied the Cox regression model to discover survival influential miRNAs in LUAD. The Cox regression model was performed using the coxph function of the Survival R package (Therneau, 2015). The clinical data of LUAD patients were retrieved by *TCGAbiolinks* (v.2.18.0) (Mounir et al., 2019) in *Bioconductor* (v3.12) release. We then performed the multivariate Cox regression model—the covariates are the expression level of the tested miRNA and the confounding clinical factors—to assess the influence of a miRNA on patient survival. Then, incorporated confounding factors are “age_at_index,” “cigarettes_per_day,” and “ajcc_pathologic_t.” In this study, we denoted the miRNAs with positive and negative coefficients in the model as harmful and protective to patient survival, respectively. The significance of the coefficient β is determined by comparing with the null model, which hypothesizes that the changes of tested miRNA expression level do not affect patient survival, and tested by a likelihood ratio test and the Wald test (Andersen and Gill, 1982; Therneau et al., 2000). The Wald test examines whether the observed regression coefficient statistically differs from zero and reported a z-score (standard score) and a *p*-value estimated by z-score for the observed regression coefficient. The miRNAs with the coefficient of *p*-value < 0.05 were recognized as the survival influential miRNAs, that is, they have a significant impact on patient survival. Consequently, 580 miRNAs were identified as survival influential miRNAs. However, we observed that approximately 68% identified survival influential miRNAs were expressed in less than 20% of patients (Figure 1B). In addition, previous studies (Concato et al., 1995; Peduzzi et al., 1995; Harrell et al., 1996) suggested that 10-20 events per predictor variable (EPV) could be appropriate for the construction of Cox regression model. In our Cox regression model, we used four predictor variables, which are expression level of the tested miRNA, and three confounding factors; thus, the proper number of events (dead samples) could be ranged from 30 to 80. Accordingly, to preserve enough coverage of patients in which the tested miRNA expressed and met the proper EPV, we decided to keep the survival influential miRNAs expressed in more than 78 dead samples, which also can cover approximately 50% samples (Figure 1B, red circles). Finally, 143 survival influential miRNAs are used in the following analysis.

Next, we used the *limma* package (v.3.46.0) (Ritchie et al., 2015) and *edgeR* (v.3.32.1) (McCarthy et al., 2012) to identify the differentially expressed (DE) isomiRs and their mRNA targets. To be consistent with the survival analysis, we used 1,522 isomiRs, which are expressed in more than 50% dead tumor samples and cover at least 41% samples, to perform differential expression analysis. The isomiR expression profiles were normalized by the Upper Quartile method and modeled with *limFit* function and *eBayes* function.

To speculate the possible functions of these isomiRs, we used the RNA-seq data of the LUAD samples as target gene expression profiles. The 3′-UTR sequences of mRNAs were also retrieved to predict the potential isomiRs’ binding targets. The RNA-seq data were first normalized with TMM, a method for estimating relative RNA production levels (Robinson and Oshlack, 2010), and using *voom* function and *eBayes* function for modeling. We found 8,557 DE genes (adjusted *p*-value = 0.01), with 3,484 upregulated and 5,073 downregulated.

### Prediction of isomiR-regulated Target Genes

We mainly use R (v4.0.3) and its packages in this research. The *Biopython* (Cock et al., 2009), which is a Python package, was used to get sequences of isomiRs and the transcript sequences. Since we only focused on the 5′ variation of isomiRs, the sequence length to where 3′ should stop was defined by the longest one for each isomiR in the whole LUAD dataset.

With the whole gene expression (444 samples) and isomiRs expression (475 samples), there are four tools used for isomiRs’ target prediction, which are: *miRanda* (v.3.3a) (Enright et al., 2003; John et al., 2004), *RNAhybrid* (v.2.1.2) (Rehmsmeier et al., 2004), *PITA* (version 6, Aug-31-08) (Kertesz et al., 2007), and TargetScan (v.7.2) (Agarwal et al., 2015); the predicted isomiR-target pairs numbers were 106,335,587, 106,439,448, 9,231,767, and 77,795,459, respectively. These algorithms follow different principles, such as the seed match, sequence complementary, the secondary structure, and free energy, thus none of them could predict the isomiR-target interactions comprehensively. Thus, we set a stringent cut-off for pairs presented in three of the four prediction tools, where only 13,091,963 pairs were kept. Finally, combined with the DE isomiR, DE gene results, and survival analysis, the protective isomiR-target pairs were classified to 10,981 (39 upregulated isomiR vs. downregulated gene) and 897 (10 dowregulated isomiR vs. upregulated gene).

### Functional Analysis of isomiRs in the Protective and Harmful Groups

The isomiR-regulated network for the functional test was selected by Spearman’s correlation, estimated by *stats:cor.test*, and visualized by *psych:cor.plo*t. Because there are many zero counts in sample-wise dimension, we compute every isomiR–isomiR correlation by intersecting non-zero value. Thus, not every correlation has the same sample number. Then, the correlation was selected by a positive coefficient (rho > 0) and top 5% by ranking both *p*-value and rho; also, only the nodes with degree > 1 were selected. For analyzing biological processes of Gene Ontology, we used *clusterProfiler:enrichGO* function (v.3.18.1), where except for the qvalueCutoff (set as 1), the other parameters were set as default. The discovered functional modules were further summarized by REVIGO (Supek et al., 2011) algorithm with a similarity ≥ 0.9 that is calculated from the Resnik algorithm (Resnik, 1999) and visualized by CirGO package (Kuznetsova et al., 2019).

## Results

### Identification of Survival Influential isomiRs in Lung Cancer

In this study, we performed the multivariate Cox regression model to identify survival influential miRNAs. The workflow is depicted in Figure 1A. To meet the appropriate EPV (Concato et al., 1995; Peduzzi et al., 1995; Harrell et al., 1996) and preserve the sufficient coverage of patients in which the tested miRNA was expressed (Figure 1B), only the identified survival influential miRNAs expressed in more than 50% dead patients (78) were kept for the following analysis. Consequently, 143 miRNAs are identified—60 and 83 miRNAs are harmful and protective, respectively (Figure 1C). Notably, 118 out of 143 survival influential miRNAs are isoforms (isomiRs) (Figure 1D). This proportion (83%) is significantly overrepresented (*p*-value = 3.21^∗^10^–5^, Fisher’s exact test). In other words, the archetype miRNAs are significantly underrepresented in these 143 survival influential miRNAs. Moreover, isomiRs associated with better prognosis of patients are significantly enriched in these 118 survival influential isomiRs (75/118, *p*-value = 8.39^∗^10^–7^, Fisher’s exact test). However, both archetype miRNAs and isomiRs are neither significantly overrepresented nor underrepresented in miRNAs associated with poor prognosis of patients. These results suggested that the survival influential isomiRs might tend to be protective and, thus, play roles as tumor suppressors and putative therapeutics for patients with lung cancer.

### Characteristics of the Identified Protective isomiRs

**FIGURE 2:**
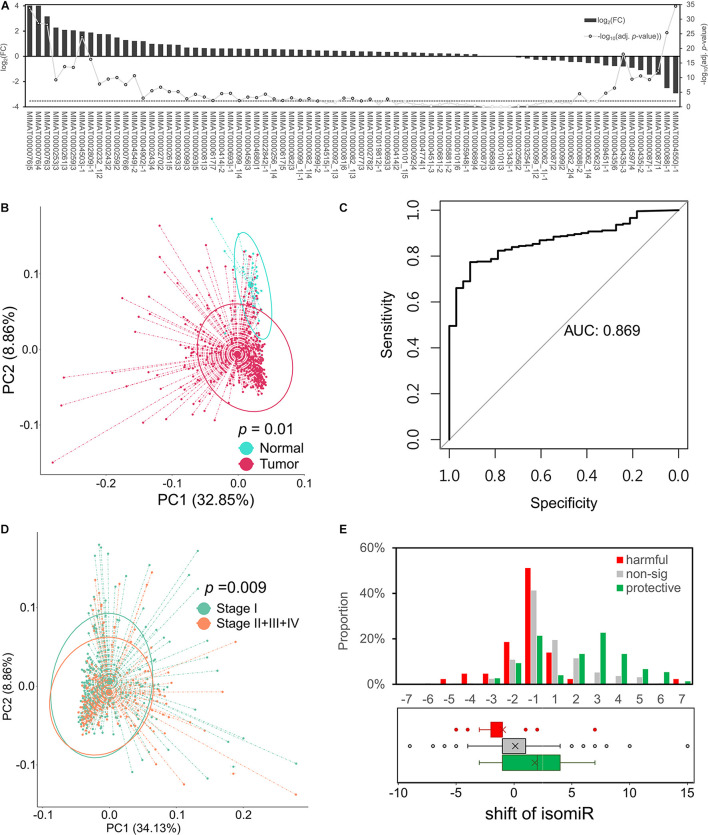
Protective isomiRs are capable to separate LUAD samples. **(A)** The differential expression pattern of the 75 identified protective isomiRs. The bars (related to the left *y*-axis) show the differential expression level of the corresponding isomiR at the *x*-axis; the line chart indicates the significance level (related to the right *y*-axis). The dashed line marks the significance level with *p*-value < 0.01. **(B)** Principal component analysis (PCA) using the 75 protective isomiRs classifies tumor and normal samples. The *p*-value is derived from permutational analysis of variance (PERMANOVA). **(C)** The ROC curve of 75 protective isomiRs by its log-transformed PC1 and PC2 to predict the total tumor samples. **(D)** PCA using the 75 protective isomiRs classifies patients with early and late stage tumors. The *p*-value is derived by PERMANOVA. **(E)** The shift distribution of isomiRs. The upper panel shows the shift distribution of protective, harmful, and insignificant isomiRs. The lower panel displays the tendency of the shift preference of the three categories of isomiRs by a boxplot.

To investigate the characteristics of these protective isomiRs, we studied their expression profiles in LUAD patients. Figure 2A shows the fold change and adj. *p*-value of these 75 protective isomiRs. Among the 75 protective isomiRs, 39 and 10 isomiRs are significantly up- and downregulated, respectively. The proportion of upregulated isomiRs (52%) is overrepresented, but the significance is moderate (right-tailed *p*-value = 0.09, Fisher’s exact test), and the proportion of downregulated isomiRs (13%) is close to randomly expected (right-tailed *p*-value = 0.44, Fisher’s exact test). This observation suggested that the identified protective isomiRs might tend to be upregulated in lung cancer. Additionally, we observed that the 75 protective isomiRs can separate tumor from normal samples significantly in the principal component analysis (PCA) (Figure 2B), but the expression profiles of completely 14,000 isomiRs and 1,522 selected isomiRs cannot (Supplementary Figure 1). Besides, using the principal components (PC1 and PC2) of 75 protective isomiRs with logistic regression model (by *glm* function in R) to predict the samples, the area under curve (AUC) reaches 0.86 (Figure 2C). These results suggested that the 75 protective isomiRs can classify the LUAD samples. The expression profiles of the 75 protective isomiRs are also distinguishable between patients of the early (I) and late (II + III + IV) stage (Figure 2D). The main difference between early and late stages is metastasis or not. Therefore, these 75 protective isomiRs might be involved in the regulation of tumor metastasis. Among these 75 protective isomiRs, 50 are positively shifted (downstream relative to archetype), and the remaining 25 are negatively shifted (upstream relative to archetype). Interestingly, we found that the protective isomiRs tend to possess positive shifts (*p*-value = 8.39^∗^10^–5^, Fisher’s exact test) and the harmful isomiRs negative shifts (*p*-value = 4.38^∗^10^–4^, Fisher’s exact test) (Figure 2E). Moreover, we observed that the protective isomiRs with downstream shift tend to be more upregulated than those with upstream shift. The average fold changes of upstream and downstream isomiRs are 1.56 and 1.01, respectively (*p*-value = 0.0564, Wilcoxon rank sum test, Supplementary Figure 2). Accordingly, these above results imply that the protective isomiRs with downstream shift might be selected to express against tumorigenesis during cancer development.

### Positive Correlation Between the Protective isomiRs

**FIGURE 3:**
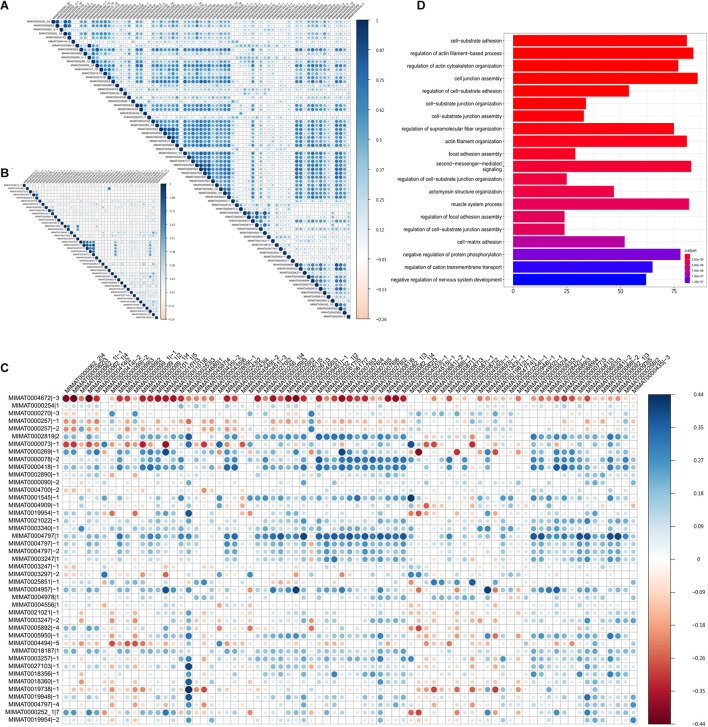
The cooperativity between survival influential isomiRs. The Spearman’s correlation between **(A)** the 75 protective isomiRs, **(B)** the 43 harmful isomiRs, and **(C)** the 75 protective and the 43 harmful isomiRs. **(D)** The top 20 significantly enriched functions regulated by the 39 highly co-expressed and upregulated protective isomiRs.

In this study, we observed the prevalent positive correlation of expression profiles between the protective or harmful isomiRs—within-group (Figures 3A,B). Additionally, among the protective isomiRs, approximately 80% of pairs are significantly and positively correlated (z-score of Spearman’s correlation coefficient > 2). This proportion is significantly overrepresented (*p*-value < 10^–271^, Fisher’s exact test). Among the harmful isomiRs, approximately 44% of pairs are significantly and positively correlated. This proportion is overrepresented, but the significance is moderate (*p*-value = 0.16, Fisher’s exact test). These observations suggest the possible cooperativity between isomiRs with a similar influence on patient prognosis. Furthermore, the cooperativity between protective isomiRs might be stronger than that between harmful isomiRs. On the other hand, we found that the significant anti-correlations among either protective or harmful isomiRs are significantly underrepresented (protective: 2%, *p*-value = 5.68^∗^10^–40^; harmful: 3%, *p*-value = 1.1^∗^10^–6^, Fisher’s exact). This observation further suggests that the isomiRs with similar influence on patient prognosis might not tend to be against each other.

On the other hand, we did not observe the concordance expression correlation between protective and harmful isomiRs (Figure 3C), suggesting that the cooperativities between protective and harmful isomiRs might not be prevalent. Furthermore, we found that the significantly negative correlations of expression profiles between protective and harmful isomiRs are significantly overrepresented (*p*-value = 1.38^∗^10^–5^, Fisher’s exact test), demonstrating the potential antagonistic relationship between protective and harmful isomiRs.

To further investigate the cooperative regulation of activated protective isomiRs, we focused on significantly 39 upregulated protective isomiRs. The functional enrichment analysis for the significantly downregulated target genes of these 39 protective isomiRs is shown in Figure 3D. We found that these target genes are significantly overrepresented in the cell migration-related functions, suggesting their roles in tumor metastasis. This observation further recapitulates the protective isomiRs’ characteristic that can classify patients with early stage tumors from late stage very well. More importantly, the above results imply that the identified upregulated protective isomiRs might repress the genes involved in tumor metastasis to benefit patient prognosis.

### The Bidirectional Regulation of Patient Prognosis

**FIGURE 4:**
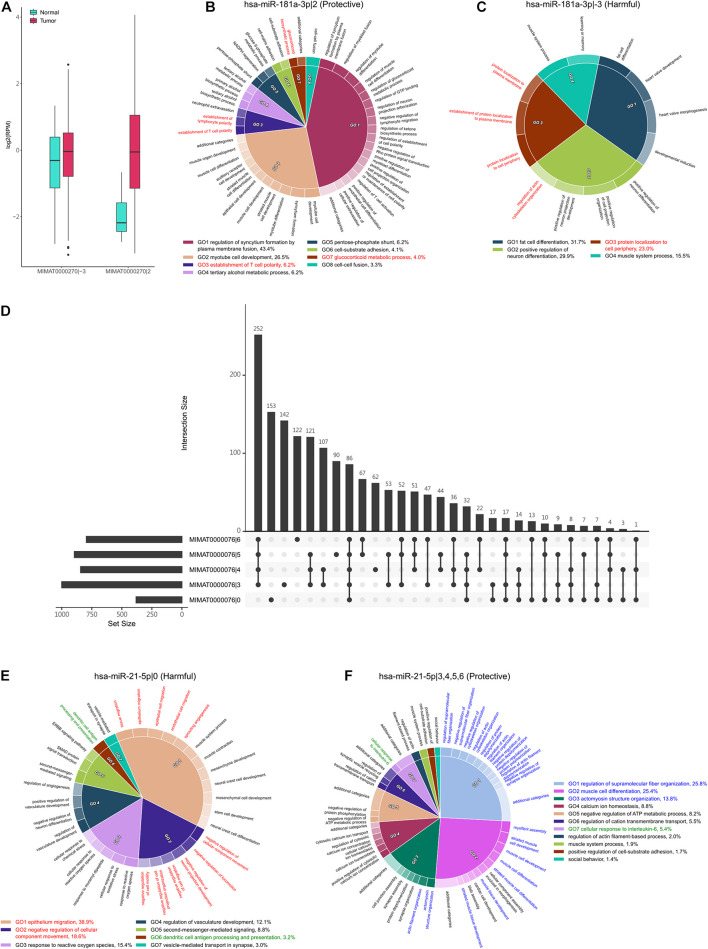
The regulatory function of hsa-miR-181a-3p|-3 and hsa-miR-181a-3p|2. **(A)** The expression of (hsa-miR-181a-3p|-3) and (hsa-miR-181a-3p|2) in tumor and normal samples. Both are upregulated in tumor samples. **(B,C)** The significantly enriched functions in which the downregulated target genes of hsa-miR-181a-3p|2 **(B)** and hsa-miR-181a-3p|-3 **(C)** are involved. The percentage is the relative significance of one functional module to others. The functional modules associated with EMT are marked by red. **(D)** The intersection of downregulated target genes between the archetype of hsa-miR-21-5p and its four protective isomiRs. The horizontal bars on the left side show the numbers of downregulated target genes of corresponding isomiRs of hsa-miR-21-5p, while the vertical bars show the intersection number. The dot represents whether the set is part of the intersection. **(E,F)** The significantly enriched functions in which the downregulated target genes of hsa-miR-21-5p|0 **(E)** and hsa-miR-21-5p|3, 4, 5, and 6 **(F)** are involved. The percentage is the relative significance of one functional module to others. The functional modules associated with metastasis, regulation of immune response, and regulation of muscle development are marked by red, green, and blue, respectively.

The interaction between isomiRs and their corresponding archetype miRNAs has been observed in lung cancer (Chan et al., 2013; Liang et al., 2020). More specifically, the isomiRs may disturb the classical regulatory network of their corresponding archetype miRNAs, even cause conflicting drug responses in cancers. In this study, we noticed, among the identified survival influential isomiRs, that MIMAT0000270|2 (hsa-miR-181a-3p|2) and MIMAT0000270|-3 (hsa-miR-181a-3p|-3) are originated from the same archetype—MIMAT0000270 (hsa-miR-181a-3p), but hsa-miR-181a-3p|2 is protective and hsa-miR-181a-3p|-3 is harmful. Interestingly, a previous study has observed that the hsa-miR-181a could function as an oncomiR or tumor suppressor in acute myeloid leukemia (Qiang et al., 2020). These observations suggest that hsa-miR-181a-3p could possess bidirectional regulation to patient prognosis. Moreover, the proportion of overlapped targets between these two isomiRs is small (0.02, Jaccard Index), emphasizing that these two isomiRs might regulate distinct downstream pathways. Additionally, these two isomiRs are upregulated in tumors (Figure 4A), suggesting that their regulatory downstream pathways might be repressed. We then performed functional enrichment analysis on their downregulated target genes separately (Figures 4B,C). We observed that the downregulated target genes of the protective hsa-miR-181a-3p|2 were significantly enriched in muscle cell or myotube functions (Figure 4B); the downregulated target genes of the harmful hsa-miR-181a-3p|-3 tend to be involved in fat cell differentiation and positive regulation of neuronal differentiation (Figure 4C). Previous studies have reported that muscle loss could be a significant predictor of patient mortality (Shiroyama et al., 2019; Rosa-Caldwell et al., 2020), supporting the protective role of hsa-miR-181a-3p|2 in lung cancer. In addition, we observed that the downregulated target genes of these two isomiRs are significantly enriched in the epithelial–mesenchymal transition (EMT)-associated functions (Figures 4B,C, marked by red). EMT is a reversible cell transition process, where cells could progressively lose the polarity, cell–cell junction, fixation, and finally turn into the mesenchymal morphology (Dongre and Weinberg, 2019). Evidence has shown that EMT was an epigenetic process, independent of DNA sequence alterations (Tam and Weinberg, 2013), and could be significant during neoplasia, contributing to the malignant progression (Mani et al., 2008; Singh and Settleman, 2010), as well as in lung cancer (O’Leary et al., 2018). For example, the harmful isomiR—hsa-miR-181a-3p|-3—may regulate “regulation of actin cytoskeleton organization” and “protein localization to plasma membrane,” pointing out that the dysregulation of its targeted genes might lose the function in fixation. On the other hand, the protective isomiR—hsa-miR-181a-3p|2—may also take part in EMT. The hsa-miR-181a-3p|2 may repress the function of the glucocorticoid metabolic process that could promote tumor cell invasion and lung metastasis (Shi et al., 2019). The other functions, such as “regulation of establishment of cell polarity” and “skeletal muscle fiber development,” may also indicate the role. The above observations suggest the potential bidirectional regulation of the hsa-miR-181a-3p. That is, these two isomiRs are derived from the same arm of pre-miRNA but demonstrate the opposite way in regulating tumor metastasis, and further patient prognosis.

In addition, miR-21 was found to be an oncogene in several cancers (Bica-Pop et al., 2018). In NSCLC, it was upregulated in patients (Yu et al., 2010; Yang et al., 2015), which also resembles our study. We observed that the archetype hsa-miR-21-5p (MIMAT0000076|0) is harmful to patients’ prognoses. However, the hsa-miR-21-5p|3, hsa-miR-21-5p|4, hsa-miR-21-5p|5, and hsa-miR-21-5p|6 are protective. Moreover, these four isomiRs are the top four upregulated isomiRs in the identified protective miRNAs. Interestingly, we found that the protective isomiRs, hsa-miR-21-5p|3, hsa-miR-21-5p|4, hsa-miR-21-5p|5, and hsa-miR-21-5p|6, have the highest intersection of target genes, while the second-highest target gene set was exclusively presented in the archetype hsa-miR-21-5p|0 (Figure 4D). This observation suggests the possible cooperativity between these four isomiRs and divergent downstream regulation from the archetype—hsa-miR-21-5p. As expected, we observed that the archetype hsa-miR-21-5p|0 is largely involved in regulating tumor metastasis-associated functions (Figure 4E, marked as red). More specifically, the downregulated target genes of hsa-miR-21-5p|0 are significantly enriched in the negative regulation of cell migration and the negative regulation of cell motility. This observation suggests that the hsa-miR-21-5p|0 may promote tumor metastasis by repressing the negative regulation of cell migration or motility. Moreover, the hsa-miR-21-5p|0 might repress the function of dendritic cell antigen processing and presentation to help tumor progression (Figure 4E, marked as green). Interestingly, the intersection of downregulated target genes between the four isomiRs, hsa-miR-21-5p|3, 4, 5, and 6, is largely significantly enriched in the function of regulation of muscle development (Figure 4F, marked as blue). This observation shows the protective roles of these four isomiRs that might co-regulate the muscle development to control muscle loss and prolong patient survival (Shiroyama et al., 2019; Rosa-Caldwell et al., 2020). In addition, we found that these four isomiRs regulate the cellular response to interleukin-6 (IL-6) (Figure 4F, marked as green). Previous studies have demonstrated the IL-6 can promote lung cancer metastasis (Gross et al., 2018; Jiang et al., 2018; Liu et al., 2020). Accordingly, these four isomiRs might prevent the patients from tumor metastasis by repressing the function of IL-6. Briefly, these above results provide a possible insight in bidirectional regulation between archetype miRNA and its isomiRs: these four isomiRs might be against the regulation of archetype hsa-miR-21-5p to repress tumor metastasis.

## Discussion

Herein, we performed a standard pipeline to identify the survival influential miRNAs to patient prognosis. We found that the isomiRs are significantly enriched in the identified survival influential miRNAs, especially for the isomiRs associated with the better prognosis of patients, that is, they are protective. These protective isomiRs might be potential therapeutics for lung cancer. Previous studies have reported that hsa-miR-21-5p was upregulated consistently across cancers (Ferracin et al., 2015) and recognized as oncomiR in lung cancer (Li and Wu, 2018; Yuan et al., 2018). Interestingly, in this study, the isomiRs originated from hsa-miR-21-5p are upregulated and associated with a better prognosis of patients: they might be tumor suppressors. IsomiRs possess different seed regions from the archetypes, suggesting that the downstream regulation could vary from an archetype to its isomiRs. Accordingly, the possible scenario is that these four isomiRs (hsa-miR-21-5p|3, hsa-miR-21-5p|4, hsa-miR-21-5p|5, and hsa-miR-21-5p|6) might evolve tumor suppressor function during cancer development. Additionally, we noticed that the protective isomiRs tend to be provided with downstream shift. Moreover, these protective isomiRs with downstream shift are more likely to be upregulated than those with upstream shift. These results suggest that the isomiRs with downstream shift might be selected during carcinogenesis to prolong patient mortality. However, further experiments are required to validate this hypothesis.

On the other hand, we observed strong cooperative interactions within isomiRs with the same prognostic effect to patients, but antagonistic interactions between protective and harmful isomiRs. Furthermore, this cooperativity can be observed from the isomiRs originated from the same precursor and shared a large proportion of regulatory target genes. For example, hsa-miR-181b-5p|−1 and −2 are both harmful isomiRs, originate from hsa-miR-181b-5p, and share significantly overrepresented target genes (Jaccard Index = 0.45; *p*-value < 0.001, Fisher’s exact test); hsa-miR-182-5p|2 and 3 are both protective isomiRs, originate from hsa-miR-182-5p, and share significantly overrepresented target genes (Jaccard Index = 0.4; *p*-value < 0.001, Fisher’s exact test). On the other hand, the antagonistic interaction can also be observed from the small shared proportion of target genes between isomiRs, for example, hsa-miR-181a-3p|2 and hsa-miR-181a-3p|-3. This observation might further imply the synergistic effect between the isomiRs exerting a similar prognostic influence on patients. That is, the combination of multiple survival influential isomiRs could benefit or worsen patient prognosis more.

In addition, we identified that hsa-miR-21-5p|0 and its four isomiRs—hsa-miR-21-5p|3, 4, 5, and 6—could play critical roles in regulating tumor metastasis. More importantly, these four isomiRs are associated with a better prognosis of patients and might be against the harmful archetype: hsa-miR-21-5p. Among the downregulated target genes of these four protective isomiRs, *HER2*, *AKT*, and *DDR2* have been observed to be aberrantly expressed in NSCLC (Tsakonas and Ekman, 2018). Moreover, *DDR2* (Discoidin domain receptor tyrosine kinase 2) plays an important role in cellular connectivity, survival, migration, and cell proliferation (Bargal et al., 2009; Fathi et al., 2018), which contributed to therapeutic target in squamous cell lung cancer (Hammerman et al., 2011). Interestingly, we found that *DDR2* is the common downregulated target gene of hsa-miR-21-5p|3, 4, and 5, but not the archetype. This exclusive regulation of oncogene might support the proposed scenario, and these four isomiRs are against the regulation of archetype to repress cancer metastasis.

In summary, we identified survival influential miRNAs for lung cancer patients. We noticed that isomiRs are more associated with patients’ mortality, especially for a better prognosis than archetype miRNAs. Moreover, the expression profiles of the protective isomiR set can be a good predictor for classifying tumor and normal samples, as well as early and late stage patients. On the other hand, we observed the cooperativity between isomiRs with similar prognostic effects to patients and possible antagonistic interaction between protective and harmful isomiRs. In the end, we gave two examples—hsa-miR-181-3p and hsa-miR-21-5p—to demonstrate that the production of isomiRs could exert a distinct downstream regulatory effect, even opposite phenotypic change: better and poor prognoses.

## Data Availability Statement

Publicly available datasets were analyzed in this study. This data can be found here: The LUAD isoform miRNA expression profile as well as transcriptome expression profile (“isoforms.quantification.txt”, “htseq.counts”) for this study can be found in the TCGA-GDC porta (https://portal.gdc.cancer.gov/). The isomiR nomenclature was followed by miRBase (http://www.mirbase.org/), where the coordinate archetype miRNA (“hsa.gff3”) downloaded from.

## Author Contributions

C-CL conceived, designed, and coordinated the study and revised the manuscript. F-MH, S-TL, M-FW, and C-CL implemented the computational method and carried out the analysis. F-MH and C-CL drafted the manuscript. All authors read and approved the final manuscript.

## Conflict of Interest

The authors declare that the research was conducted in the absence of any commercial or financial relationships that could be construed as a potential conflict of interest.

## Publisher’s Note

All claims expressed in this article are solely those of the authors and do not necessarily represent those of their affiliated organizations, or those of the publisher, the editors and the reviewers. Any product that may be evaluated in this article, or claim that may be made by its manufacturer, is not guaranteed or endorsed by the publisher.

## Footnotes

1
https://portal.gdc.cancer.gov/
