# Supplementary material for: Identification and Elucidation of the Protective isomiRs in Lung Cancer Patient Prognosis
Source: Front Genet. 2021 Sep 13;12:702695. doi: 10.3389/fgene.2021.702695 (PMC8474875; doi:10.3389/fgene.2021.702695)
Supplement: Supplementary file 1 [file Data_Sheet_1.PDF]

frontiers

Supplementary Material

Supplementary Figures

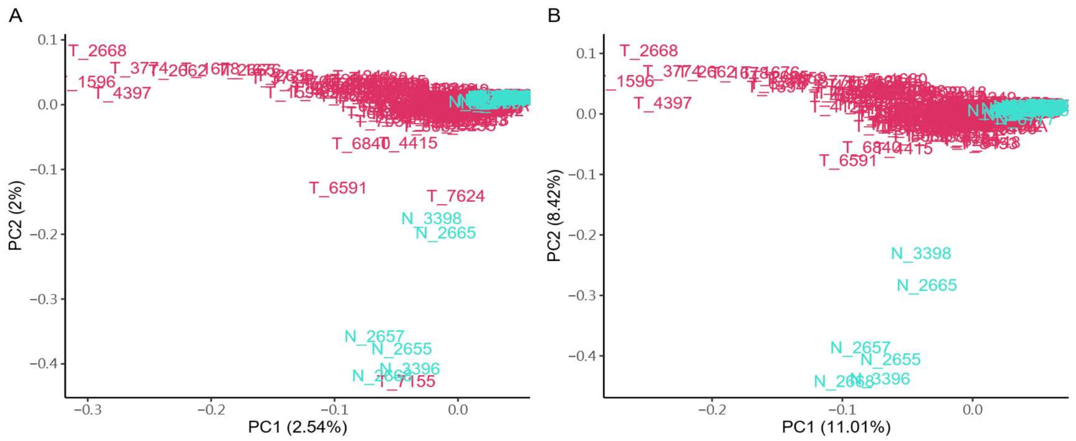

Supplementary Figure 1: Principal component analysis of isomiRs in LUAD 33 normal and 442 tumor LUAD samples. **(A)** PCA of 14000 isomiRs **(B)** PCA of 1522 isomiRs expressed in at least 50% dead patients.

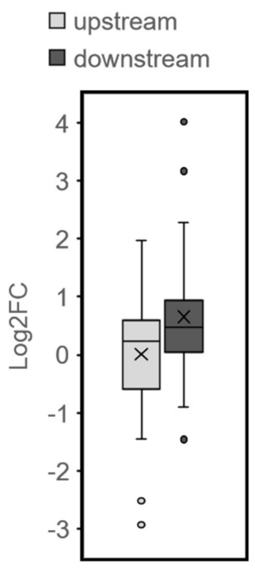

Supplementary Figure 2: Log2 fold change of protective isomiRs with the upstream and downstream shift.
